# Decorin Knockdown Improves Aged Tendon Healing by Enhancing Recovery of Viscoelastic Properties, While Biglycan May Not

**DOI:** 10.1007/s10439-024-03612-y

**Published:** 2024-11-29

**Authors:** Christelle Darrieutort-Laffite, Stephanie N. Weiss, Courtney A. Nuss, Joseph B. Newton, Jeremy D Eekhoff, Louis J. Soslowsky

**Affiliations:** 1 McKay Orthopaedic Research Laboratory, University of Pennsylvania, Philadelphia, Pennsylvania USA; 2 Regenerative Medicine and Skeleton, RMeS, UMR 1229, Nantes Université, CHU Nantes, INSERM, F-44000 Nantes, France; 3 Department of Bioengineering, University of Pennsylvania, Philadelphia, PA USA; 4 Rheumatology Department, Nantes University Hospital, 1 place Alexis Ricordeau, 44000 Nantes, France

**Keywords:** Tendon, Injury, Healing, Aging, Proteoglycans

## Abstract

The objective of the study was to determine the specific roles of decorin and biglycan in the early and late phases of tendon healing in aged mice. Aged (300 day-old) female wildtype (WT), *Dcn*^*flox/flox*^ (I-*Dcn*^*-/-*^), *Bgn*^*flox/flox*^ (I-*Bgn*^*-/-*^), and compound *Dcn*^*flox/flox*^*/Bgn*^*flox/flox*^ (I-*Dcn*^*-/-*^*/Bgn*^*-/-*^) mice with a tamoxifen (TM) inducible Cre underwent a bilateral patellar tendon injury (PT). Cre excision of the conditional alleles was induced at 5 days (samples collected at 3 and 6 weeks) or 21 days post-injury (samples collected at 6 weeks). Scar tissue area, collagen architecture, gene expression and mechanical properties were assessed during re-establishment of tendon architecture after injury. Fibril diameter distribution was impacted by both decorin and biglycan knockdown at 3 and 6 weeks compared to WT. Although early healing appeared to be delayed in the I-*Bgn*^*-/-*^ tendons (larger scar tissue area at 3 weeks), no differences in failure properties were detected. By 6 weeks, in the I-*Dcn*^*-/-*^ tendons, we observed a better recovery of viscoelastic properties compared to the WT tendons (reduced stress relaxation and increased dynamic modulus) when the knockdown was induced early. This could be explained by the increased expression of other matrix proteins, such as elastin whose gene expression was increased at 3 weeks in the I-*Dcn*^*-/-*^ tendons. Despite an impact on collagen fibrillogenesis, decorin and/or biglycan knockdown did not produce a detectable effect on quasi-static properties after patellar tendon injury. However, early decorin knockdown resulted in better recovery of viscoelastic properties. Mechanisms underlying this result remained to be clarified in further studies.

**Supplementary Information:**

The online version contains supplementary material available at 10.1007/s10439-024-03612-y.

## Introduction

Tendon is characterized by a highly organized matrix that allows the transmission of forces from muscle to bone. Tendon injuries are a frequent clinical problem in athletic and non-athletic patients [1–3]. They are associated with significant pain and disability and represent a considerable burden on the health care system [4]. Advanced age reduces the regenerative capacity of tendons and increases the susceptibility to injuries [5, 6]. For example, rotator cuff tendon abnormalities increase from 13% before the age of 40 to 30% between 60 and 69, and rise to 62% over 80 [2]. Tendon aging is associated with modifications of the stem cells niche involving cell–cell communication, extracellular matrix, oxidative stress, and vascularity combined with age-associated changes of hormonal and metabolic signals [5]. As a consequence, changes related to aging impacts recovery of mechanical properties after injury [7, 8].

Decorin and biglycan are both small leucine rich proteoglycans (SLRPs) which are highly expressed throughout the tendon extracellular matrix. They compete for the same binding site on collagen I fibrils and regulate matrix assembly and structure. However, their temporal expression profile is different; biglycan is most highly expressed during the early phases of development while decorin is highly expressed throughout aging [9]. In 485 day-old mice, knockout of biglycan resulted in impaired elastic properties when compared to WT tendons [10]. In contrast, the absence of decorin expression decreased the effects of tendon aging [11].

After injury, the effects of SLRP knockouts were different according to age. In 120 day-old mice, biglycan-null mice were deficient in early stage healing while decorin-null mice were deficient in late-stage healing [12]. In contrast, patellar tendons (PT) from biglycan-null and decorin-null 270 day-old mice both had altered early stage healing [13]. These findings suggested that the impact of SLRPs on tendon healing varies by age. The main limitation of these previous studies was the potential for compensation by the remaining extracellular matrix (ECM) constituents since the SLRPs were knocked out throughout development, making it difficult to isolate the specific effects of each SLRP on tendon healing. Knockout of one proteoglycan could cause upregulation of other matrix components that play a similar mechanical role generating confounding factors [14]. Recently, inducible knockdown models showed, in P120 mice, that decorin knockdown at the time of injury or later did not affect tendon healing and that the most impactful role of biglycan in restoring tendon mechanical properties occurred in late‐stage healing [15].

To better understand the role of decorin and biglycan in the injury response of aged mice, inducible knockdown mouse models were used in this study. They allow genetic inactivation of decorin and biglycan expression in the aged tendon while maintaining normal expression during development. The objective of the study was to determine the specific roles of decorin and biglycan in the early and late phases of tendon healing using inducible knockdown aged mice (300-day old)  by analyzing the effects of their knockdown (decorin, biglycan or both) on tendon morphology, collagen architecture, mechanical properties and gene expression during re-establishment of tendon architecture after injury. Three hundred-day old mice were considered as an aged group because of the decline in mechanical properties of the tendons compared to mature (150-day) mouse tendons combined with the similarities between the survival curves of mice at this age and 60–65-year-old women [16]. Based on previous data obtained with conventional knockout models [12, 13, 17], we hypothesized that decorin knockdown would result in more pronounced alterations of healing compared to biglycan knockdown. We also hypothesized that biglycan knockdown would have a greater impact with early inactivation compared to inactivation later during healing.

## Materials and Methods

### Animals, Injury Model and Sample Collection

**Fig. 1:**
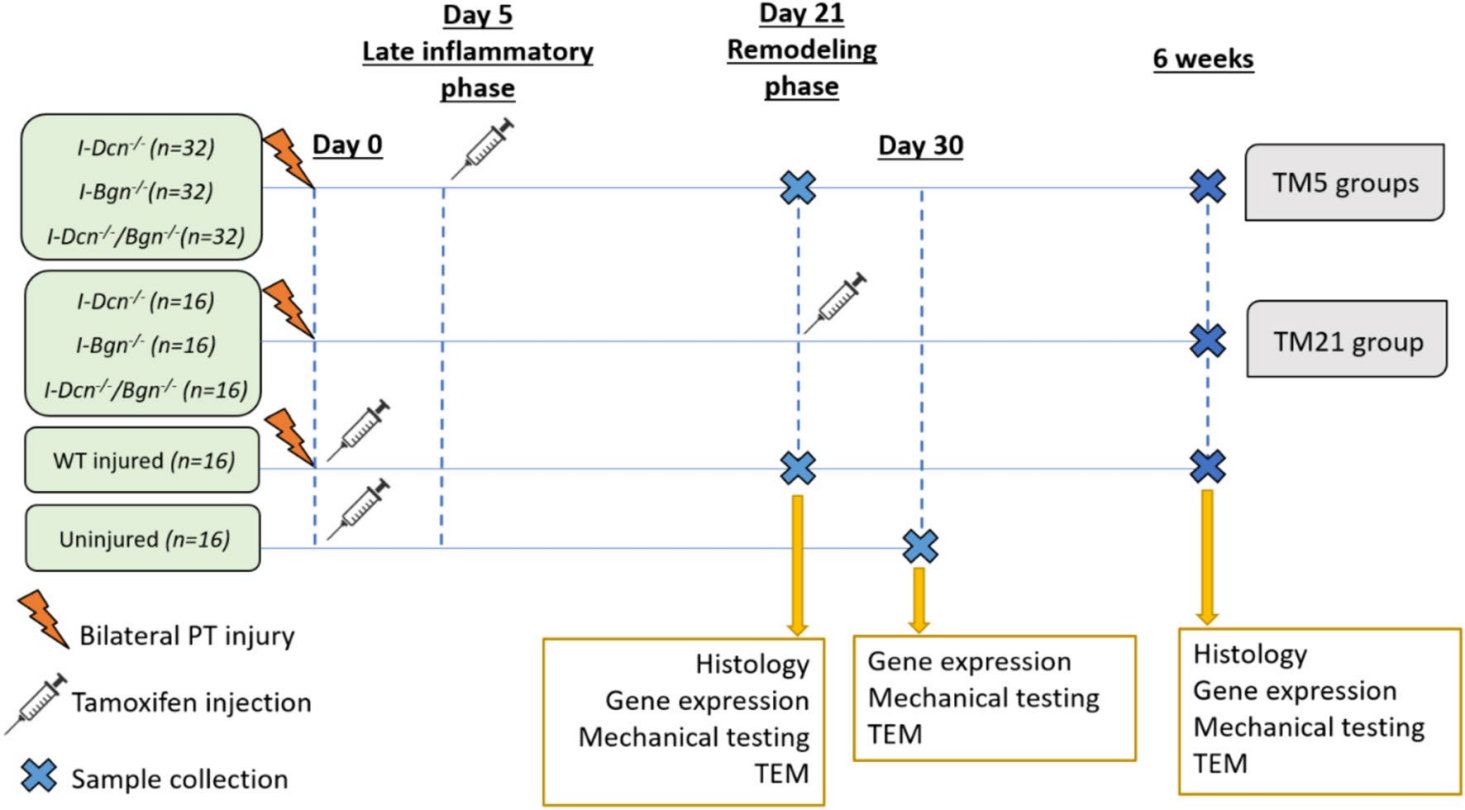
Study design (*TM5* induction of knockdown at 5 days post-injury; *TM21* induction of knockdown at 21 days post-injury; *WT* wilt type)

Female wild-type (*n* = 32;16 injured and 16 uninjured), *Dcn*^*flox/flox*^ (I-*Dcn*^*-/-*^, *n* = 48), *Bgn*^*flox/flox*^ (I-*Bgn*^*-/-*^, *n* = 48), and compound *Dcn*^*flox/flox*^*/Bgn*^*flox/flox*^ (I-*Dcn*^*-/-*^*/Bgn*^*-/-*^, *n* = 48) mice with a tamoxifen (TM) inducible Cre, (B6.129-Gt (ROSA)26Sortm1(cre/ERT2)Tyj/J, Jackson Labs) were used (IACUC approved). Aged mice (300 days) underwent bilateral patellar tendon (PT) injury, as previously described [18]. Briefly, under sterile conditions, the patellar tendon was exposed after a skin incision near the knee. Longitudinal incisions were made on both sides of the tendon allowing the insertion of a rubber coated backing behind the tendon. Then, a full thickness, partial width defect was made at the center of the PT using a 0.75 mm biopsy punch. The mice returned to cage activity after the surgery. Following injury, Cre excision of the conditional alleles was induced at 5 days (TM5 groups) during the late inflammatory phase of healing or at 21 days (TM21 groups) during the remodeling phase via two consecutive daily intraperitoneal injections of tamoxifen (Sigma, T5648) (50 mg/kg body weight). Mice from inducible knockdown genotypes were sacrificed at 3 or 6 weeks post-injury for the TM5 groups while mice from the TM21 groups were sacrificed at 6 weeks post-injury. All wild-type mice received Tamoxifen injections at 300 days. Injured wild-type controls (WT) were sacrificed 3 and 6 weeks after injury, while uninjured wild-type controls (uninjured) were sacrificed 30 days after tamoxifen injection (Figure 1).

### Histology

Knees were fixed in 10% neutral buffered formalin for 48 hours, decalcified in 5% formic acid (Immunocal, Stat Labs) and embedded in paraffin (*n* = 4 knees/group). Transverse sections with a thickness of 10 µm were obtained every 200 µm along the injury and stained with Toluidine Blue. The ratio of scar tissue area to tendon area was quantified using a custom MATLAB script to manually trace the boundaries of the scar and the whole tendon on 6 slides per sample. Scar area is expressed as a percentage of whole tendon area (6-slide average). Researchers were blinded to genotype and time point.

### Transmission Electron Microscopy (TEM)

Patellar tendons were fixed in Karnovsky’s Fixative immediately after sacrifice, post-fixed and stained in 1% osmium tetroxide, dehydrated in ethanol, separated into proximal and distal regions, and embedded in epon resin (*n* = 4 tendons/group). Tendons were then sectioned in the transverse plane with an ultramicrotome (60–80 nm thick) and stained with uranyless (EMS22409) followed by 1% phosphotungstic acid. Grids were examined at 60,000x magnification using a JEOL 1010 transmission electron microscope. Ten images were captured in the injury region and the diameter of collagen fibrils was measured using a custom MATLAB script. Images were binarized using Otsu’s thresholding method and adjacent fibrils were distinguished using watershed segmentation. Fibril diameter was determined to be the minor diameter of the ellipse fit to each fibril. Diameter measurements were visually confirmed by overlaying fit ellipses over the raw image [15]. Researchers were blinded to genotype and time point.

### Mechanical Testing

The patellar tendon-bone complex from one limb of each animal was dissected (*n* = 8 tendons in the WT injured group; *n* = 16 tendons in the TM5, TM21 and uninjured group). Care was taken not to disturb scar tissue to avoid damaging the tendon and to retain native properties. Tendons were stamped to a width of 0.75 mm to increase the involvement of the injured region in the measured mechanical properties. Cross-sectional area was measured before and after stamping using a laser displacement sensor along with a x–y linear stage and two linear variable differential transformers to create a 3D reconstruction of the tendon volume [15]. Verhoeff’s stain lines were applied at 0, 1, 2, and 3 mm from the tibial insertion to allow optical strain tracking of the injured tendon. The tibia was potted in polymethyl methacrylate and the patella was gripped in a custom fixture. Then, the tendon was mounted within a mechanical testing system (5848, Instron; Norwood, MA) and submerged in a phosphate buffered saline bath at 37 °C. After a pre-load at 0.03N, the applied testing protocol consisted of 10 cycles of preconditioning (sinusoidal oscillations with 0.5% engineering strain amplitude centered at 1% strain), 5 minutes of recovery at 0% strain, stress relaxations at 3, 4, and 5% strains held for 10 minutes followed by frequency sweeps at 0.1, 1, 5, and 10 Hz at each strain level, 5 minutes rest at 0% strain, and a quasi-static ramp-to-failure (0.1% strain per second). Percent relaxation was quantified for each stress-relaxation. Dynamic modulus and phase angle delta (tan δ) were computed by fitting sinusoidal functions to engineering stress and engineering strain as a function of time for each frequency sweep at each strain level. Maximum engineering stress and elastic modulus were assessed during the ramp-to-failure. Images were captured at 15 fps and 1600x1200 resolution using a camera (Basler acA1920-155um) and telephoto lens (Nikon AF Micro-Nikkor ED 200 mm f/4 D IF). Lagragian strain was calculated by tracking four distinct points on the Verhoeff’s stain lines using rigid image registration [15, 18]. Dynamic collagen fiber realignment was also assessed in the injured region throughout the ramp-to-failure test using a rotating crossed polarizer setup and compared between groups at discrete engineering strains [19]. Researchers were blinded to genotype and time point.

### Gene Expression Analysis

Patellar tendons were removed and snap frozen in liquid nitrogen within 7 minutes of sacrifice (*n* = 4 tendons/group). Samples were then homogenized in TRIzol (Invitrogen) using a pestle. Total RNA was extracted using the Direct-zol RNA Microprep kit (Zymo, R2062), and RNA was reverse-transcribed using a High Capacity cDNA RT kit (ThermoFisher). Then, cDNA underwent 15 cycles of pre-amplification with selected Taqman Gene Expression Assays (Preamp Master Mix, PN 100-5744, Fluidigm). Pre-amplified cDNA was finally loaded into a Fluidigm 96.96 Dynamic Array and the expression of 96 selected genes was assessed to study collagens, non-collagenous matrix components, genes involved in matrix remodeling, cell differentiation markers, interactions between cells and extracellular matrix, signaling and inflammation, cell proliferation and Toll-like receptors. Resultant cycle threshold (Ct) values were normalized to the invariant controls (*ABL1* and *RPS17*) and expressed as 2^ΔCt^. Researchers were blinded to genotype and time point.

### Statistics

Sample sizes for each assay were determined *a priori* using power analysis (*α* = 0.05, power = 0.8) or was based on previous experiments. The effect size f was 0.43 for mechanical testing data, our primary outcome, and 0.97 for gene data. Power analyses could be performed on data expected to be non-normal (fibril diameter distribution and scar area data) without underlying assumptions about their respective distributions. The type of statistical testing (i.e., parametric vs. non-parametric) was determined based on the normality of the data. A Kruskal-Wallis test was used to compare the scar tissue area between groups at each time points. Fibril diameter distributions were compared using a Kolmogorov‐Smirnov test. One-way ANOVAs with Bonferroni corrections were conducted to compare mechanical properties and gene expression between groups. A mixed-effects model was used to compare collagen realignment across genotypes and strains. Significance was set at *p* < 0.05, and differences were considered trending at *p* < 0.10.

## Results

**Fig. 2:**
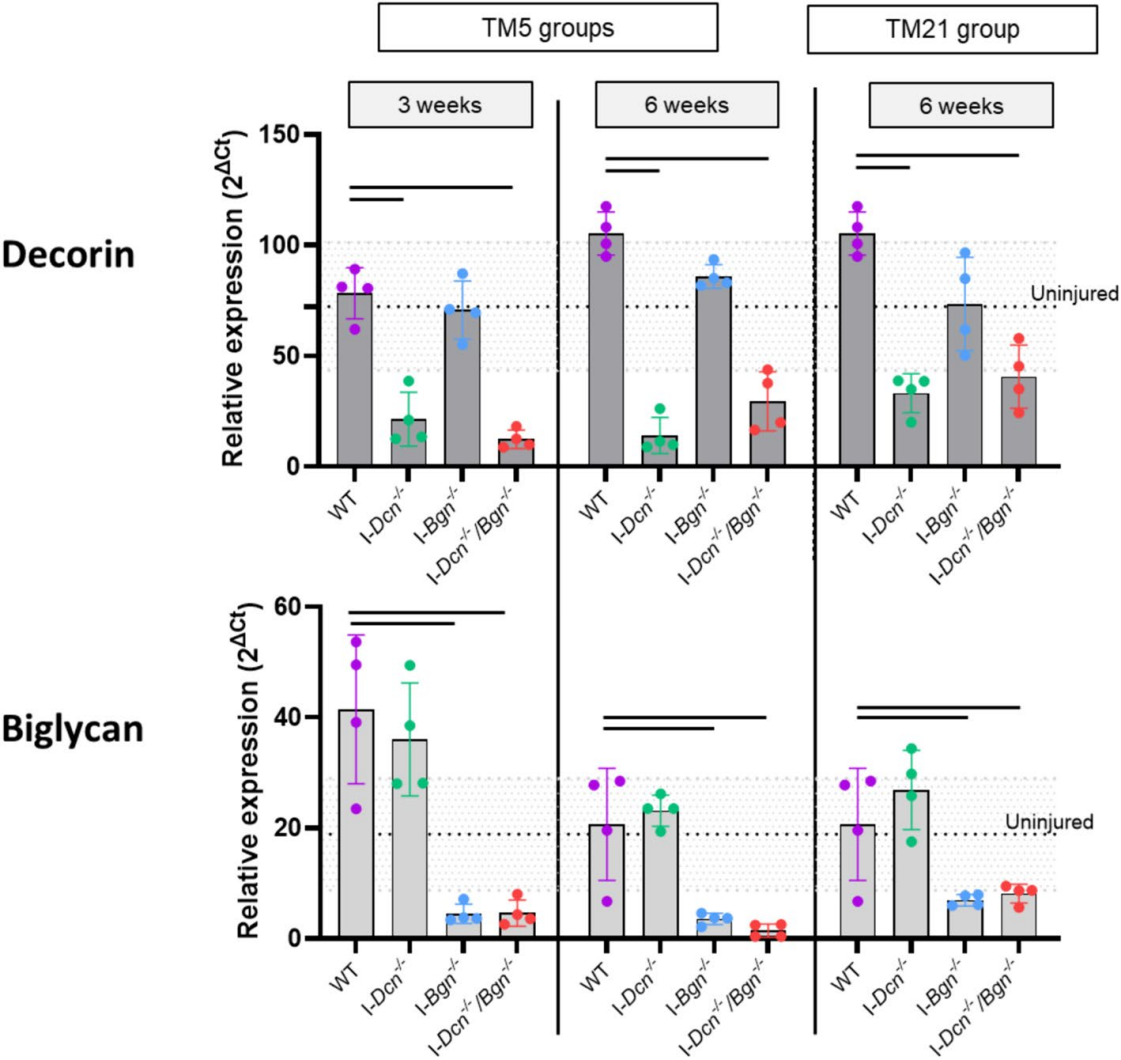
Gene expression of decorin and biglycan after the induction of the knockdown. After tamoxifen injection, we observed substantial decrease in decorin and biglycan expression at 3 and/or 6 weeks compared to WT injured samples related to the corresponding genotype, demonstrating an effective knockdown of both genes (*n* = 4/group). Errors bars represent standard deviation. Dotted lines represent average mRNA level of expression in uninjured tendons and grey dotted lines limits represent standard deviation of the uninjured group. Significant differences (*p* < 0.05) between genotypes are represented by solid lines.

- 1. Decorin and biglycan gene expression was significantly reduced after tamoxifen induction (Fig. 2).

As expected, tamoxifen injection at 5 days and 21 days post-injury significantly reduced Decorin expression at 3 weeks and/or 6 weeks in the I-*Dcn*^*-/-*^ and the I-*Dcn*^*-/-*^*/Bgn*^*-/-*^ tendons. In the I-*Bgn*^*-/-*^ and the I-*Dcn*^*-/-*^*/Bgn*^*-/-*^ tendons, a significant reduction in biglycan expression was observed at 3 weeks and/or 6 weeks post-injury after TM5 or TM21 tamoxifen injection, respectively. In addition, no compensatory change in Dcn expression in the I-*Bgn*^*-/-*^ tendons or in Bgn expression in the I-*Dcn*^*-/-*^ tendons was observed.

At 3 weeks, there was a trend towards an increase in biglycan expression in WT injured tendons compared to uninjured samples (*p* = 0.057) while decorin expression level was not significantly different than uninjured tendons in WT injured tendons at all time points.

**Fig. 3:**
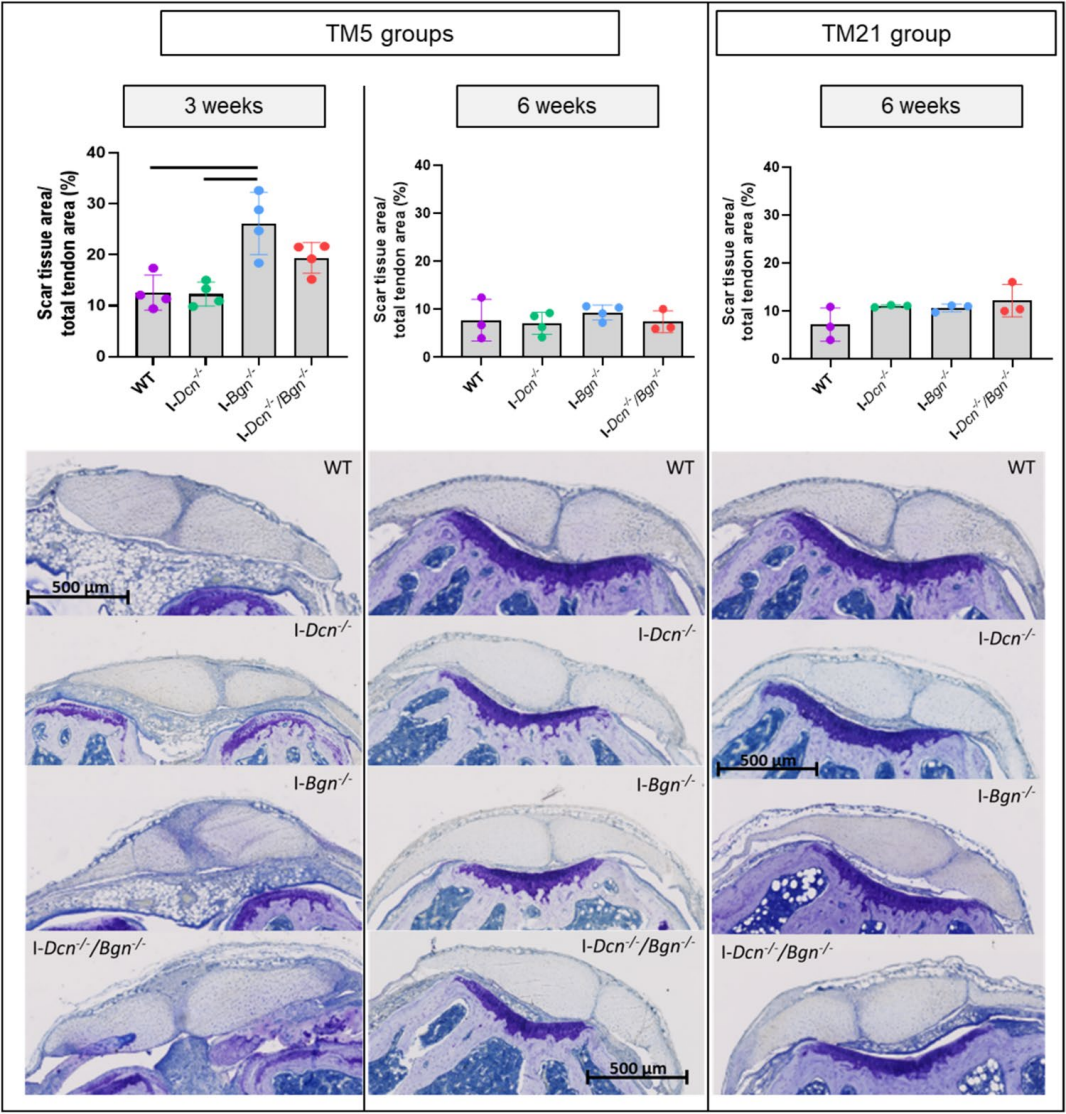
Scar tissue area assessed by histology (Toluidine Blue staining). A larger scar area was observed at 3 weeks after biglycan knockdown, but this difference was no longer observed at 6 weeks (*n* = 3–4/group). Scar area is expressed as a percentage of whole tendon area (the 6-slide average of each tendon is plotted as an individual datum). Errors bars represent standard deviation. Representative images from all groups at each time points are provided. Significant differences (*p* < 0.05) between genotypes are represented by solid lines.

- 2. Tendon healing appeared to be delayed after early biglycan knockdown (Fig. 3)

With knockdown at 5 days post-injury, the scar tissue area was larger in the I-*Bgn*^*-/-*^ tendons compared to the I-*Dcn*^*-/-*^and WT tendons after 3 weeks. At 6 weeks, this difference was no longer detectable. When SLRPs were inactivated 21 days after injury, no impact on healing was observed with similar scar tissue area in all groups.

**Fig. 4:**
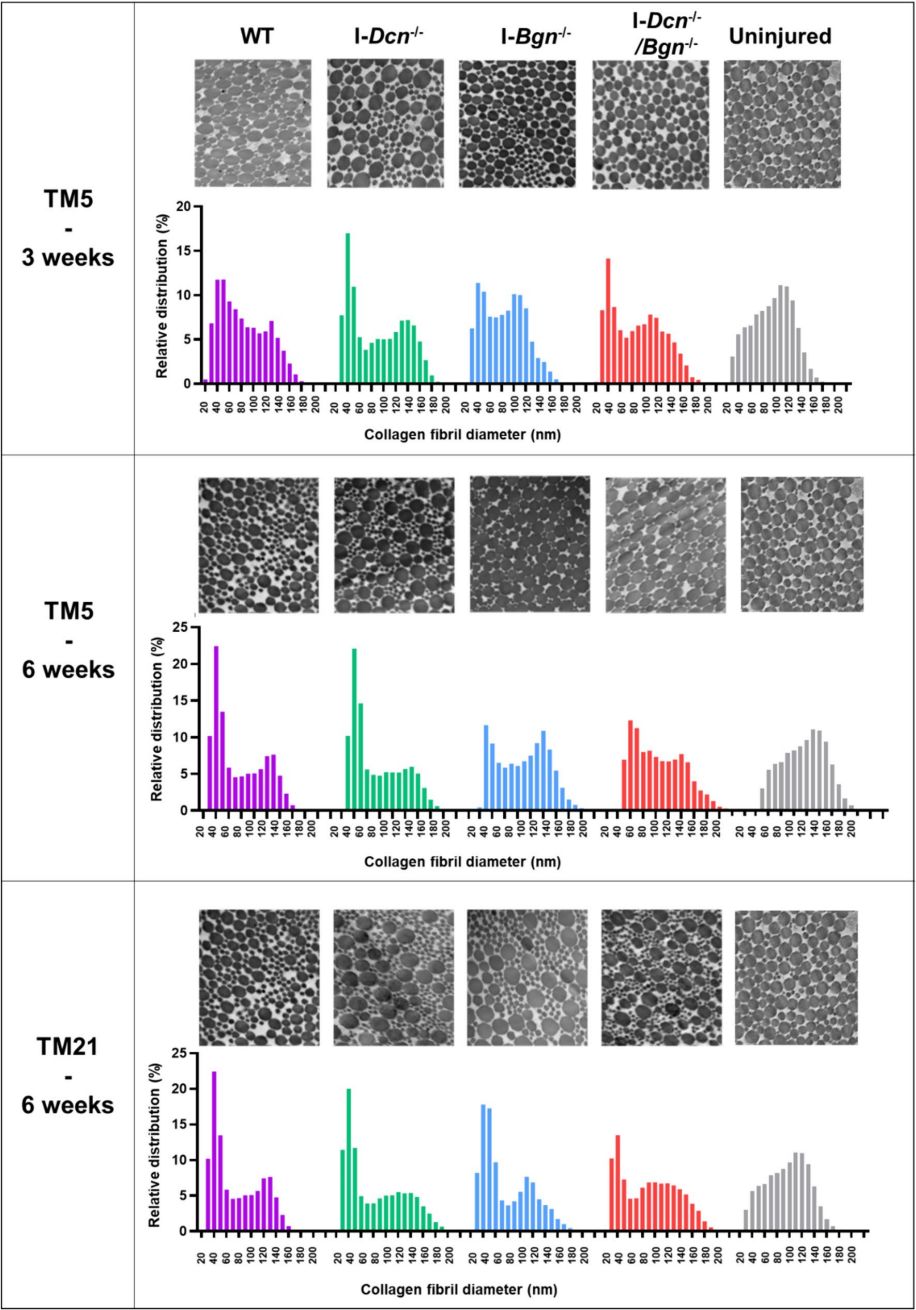
Assessment of tendons by transmission electron microscopy (TEM) at different timepoints. At 3 weeks post-injury, the proportion of small fibrils (40 nm) was higher in both the I-*Dcn*^*-/-*^ and I-*Dcn*^*-/-*^*/Bgn*^*-/-*^ tendons while I-*Bgn*^*-/-*^ tendons exhibited more widespread fibril diameter distributions. Representative images and fibril diameter distributions are presented for all groups (*n* = 4 tendons/group).

- Fibril diameter distributions were different between groups at each timepoint (Fig. 4).

Collagen fibril morphology within the healing region was evaluated using TEM. At 3 and 6 weeks post-injury, an increase in small diameter fibers (around 40 nm) was observed in all groups (Figure 4) compared to uninjured tendons which exhibit the expected bimodal distribution of normal tendon. At each time point, fibril diameter distributions were different between groups (Figure 4, p < 0.0001, Kolmogorov-Smirnov test). At 3 weeks post-injury, the proportion of 40 nm fibrils was higher in both the I-*Dcn*^*-/-*^ and I-*Dcn*^*-/-*^*/Bgn*^*-/-*^ tendons while the WT and I-*Bgn*^*-/-*^ tendons exhibited more frequent medium-sized fibers (between 60 and 120 nm). In TM5 groups, at 3 weeks as well as 6 weeks post-injury, interquartile range was higher in I-*Bgn*^*-/-*^ tendons suggesting more widespread distribution of fibril diameters (supplementary table 1). In the same TM5 groups at 6 weeks post-injury, the higher proportion of small fibers was persistent in the I-*Dcn*^*-/-*^ group although the fibril diameter distribution profile in the I-*Dcn*^*-/-*^*/Bgn*^*-/-*^ tendons was closer to the I-*Bgn*^*-/-*^ tendons. Finally, there were no morphological abnormalities of the fibers.

**Fig. 5:**
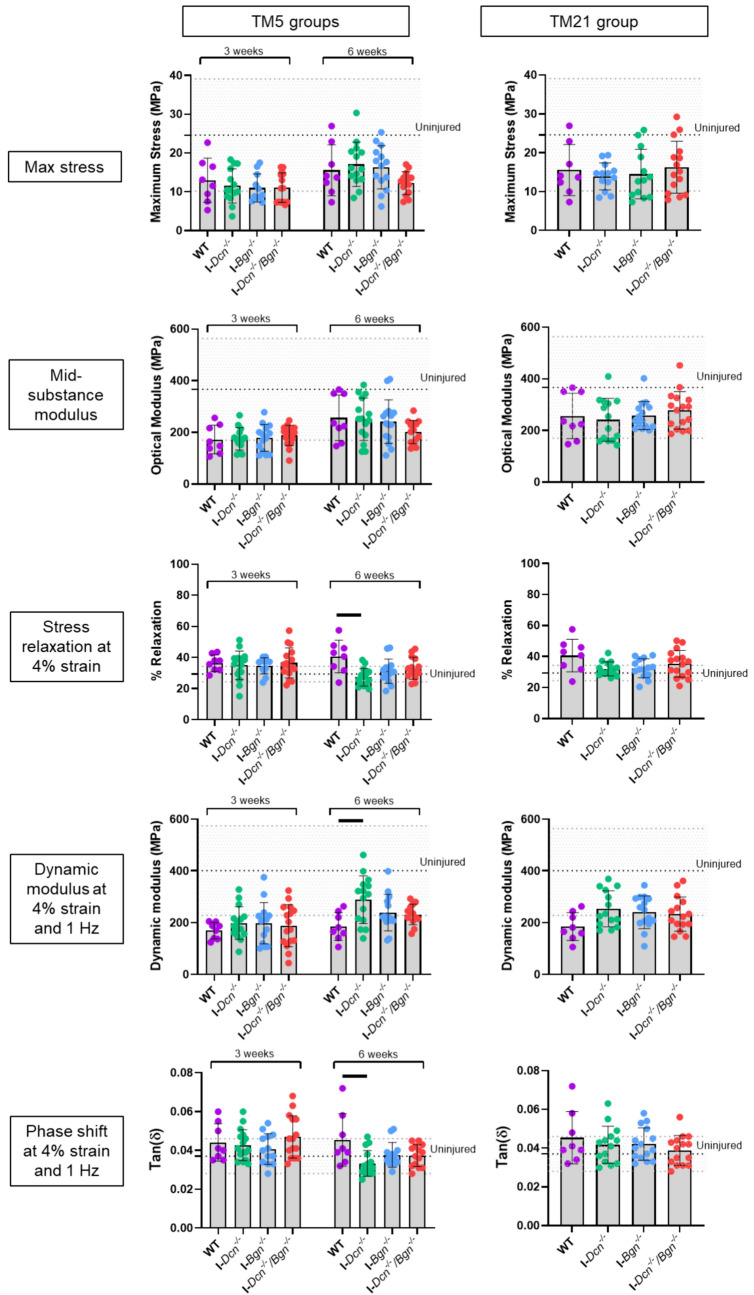
Mechanical properties of tendons after Dcn and/or Bgn knockdown at 5 or 21 days post-injury. While failure stress was not detectably different between genotypes, decorin knockdown at 5 days post-injury was associated with a decreased stress relaxation, an increased dynamic modulus and decreased tan δ at 6 weeks compared to WT, suggesting a better recovery of viscoelastic properties (*n* = 8 for WT and 12-15 for other groups). Viscoelastic data at other strains (3 and 5% strains, (Supplementary Fig.2)) and frequencies yielded similar results (data not shown). Errors bars represent standard deviation. Dotted lines represented average values for uninjured samples and grey dotted lines limits represent standard deviation of this group. Significant differences (*p* < 0.05) between genotypes are represented by solid lines.

- Viscoelastic properties recovered better in I-Dcn^-/-^ tendons after early knockdown (Fig. 5)

To assess impact of SLRPs knockdown on healing tendon function, we tested recovery of mechanical properties over time. After dissection, there was no significant difference in cross-sectional area between groups (Supplementary figure S1). As expected, at 3 weeks after injury, max stress and mid-substance modulus were reduced in all groups compared to uninjured samples but no differences were observed in the I-*Dcn*^*-/-*^*,* I-*Bgn*^*-/-*^, and I-*Dcn*^*-/-*^*/Bgn*^*-/-*^ tendons compared to WT tendons (Figure 5).

Tendon viscoelastic properties were evaluated with a series of stress relaxations at 3%, 4%, and 5% strain to measure percent relaxation, and frequency sweeps at the same strains at 0.1, 1, 5, and 10 Hz to evaluate the dynamic modulus and phase shift (tan δ). The I-*Dcn*^*-/-*^ tendons exhibited a decreased stress relaxation, an increased dynamic modulus associated, and decreased tan δ at 6 weeks compared to wildtype when the knockdown was induced 5 days after the injury, suggesting a better recovery of viscoelastic properties (Figure 5, Supplementary figure S2).

Inactivation of decorin and/or biglycan at 21 days post-injury did not produce a detectable impact on viscoelastic parameters (Figure 5).

**Fig. 6:**
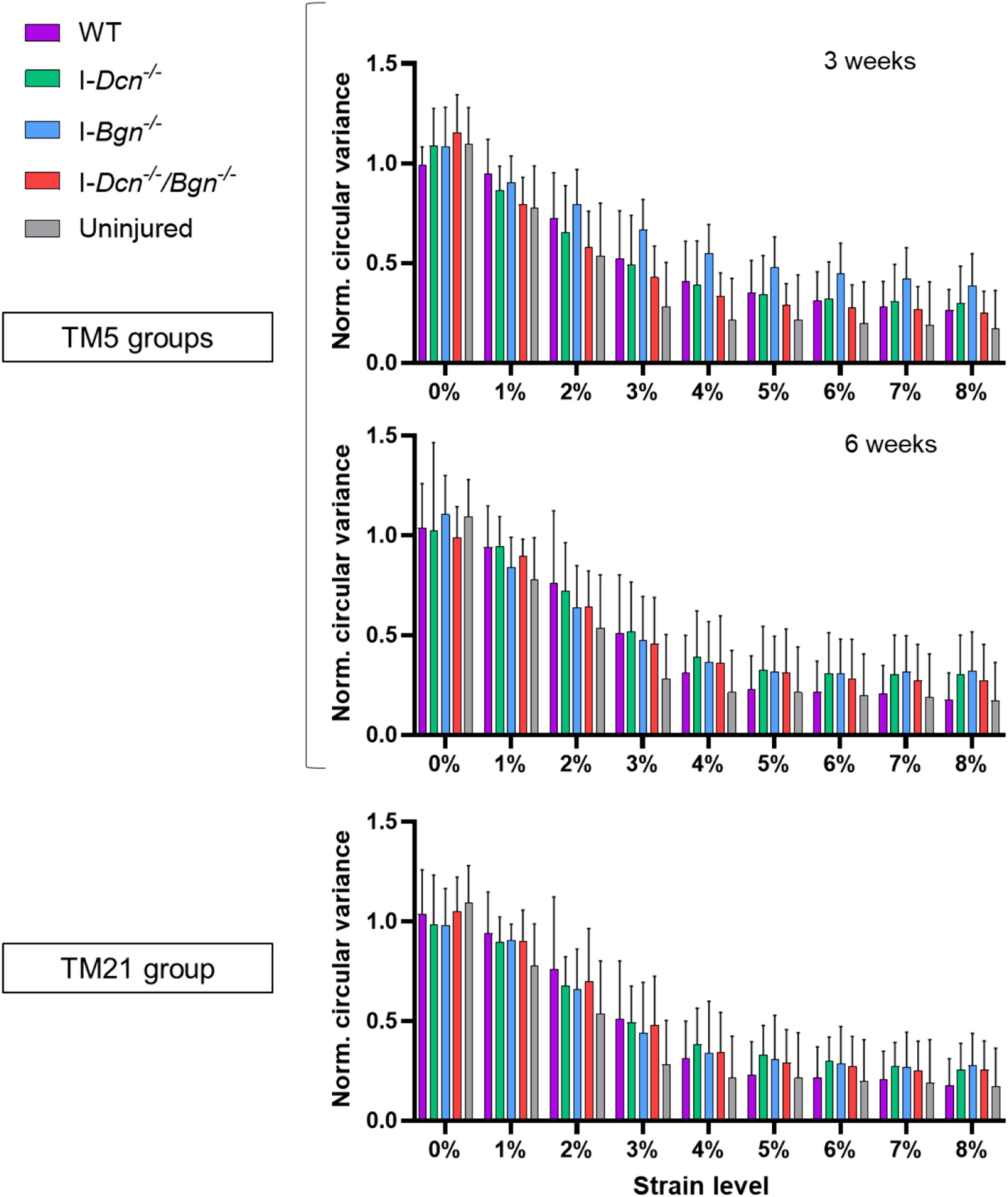
Dynamic fiber realignment during the ramp-to-failure at the injury region. Reduced normalized circular variance decreased with increasing strain, this suggesting an increase in collagen realignment. However, no significant differences were observed between genotypes whether the knockdown was induced at 5 days or 21 days after the injury (*n* = 8 in the WT group, *n* = 12–15 in the other groups). Error bars represent standard deviation.

Finally, looking at dynamic fiber realignment during the ramp-to-failure at the injury region, there was an increase in collagen realignment with increasing strain level. There was a trend toward a decrease in fiber realignment (i.e., higher circular variance) at 4, 5, and 6 % strain in the I-*Bgn*^*-/-*^ tendons compared to I-*Dcn*^*-/-*^ tendons at 3 weeks; however, no significant differences in fiber realignment were observed after decorin and/or biglycan knockdown compared to the WT group (Figure 6).

- 5. qPCR of tendons during healing

**Fig. 7:**
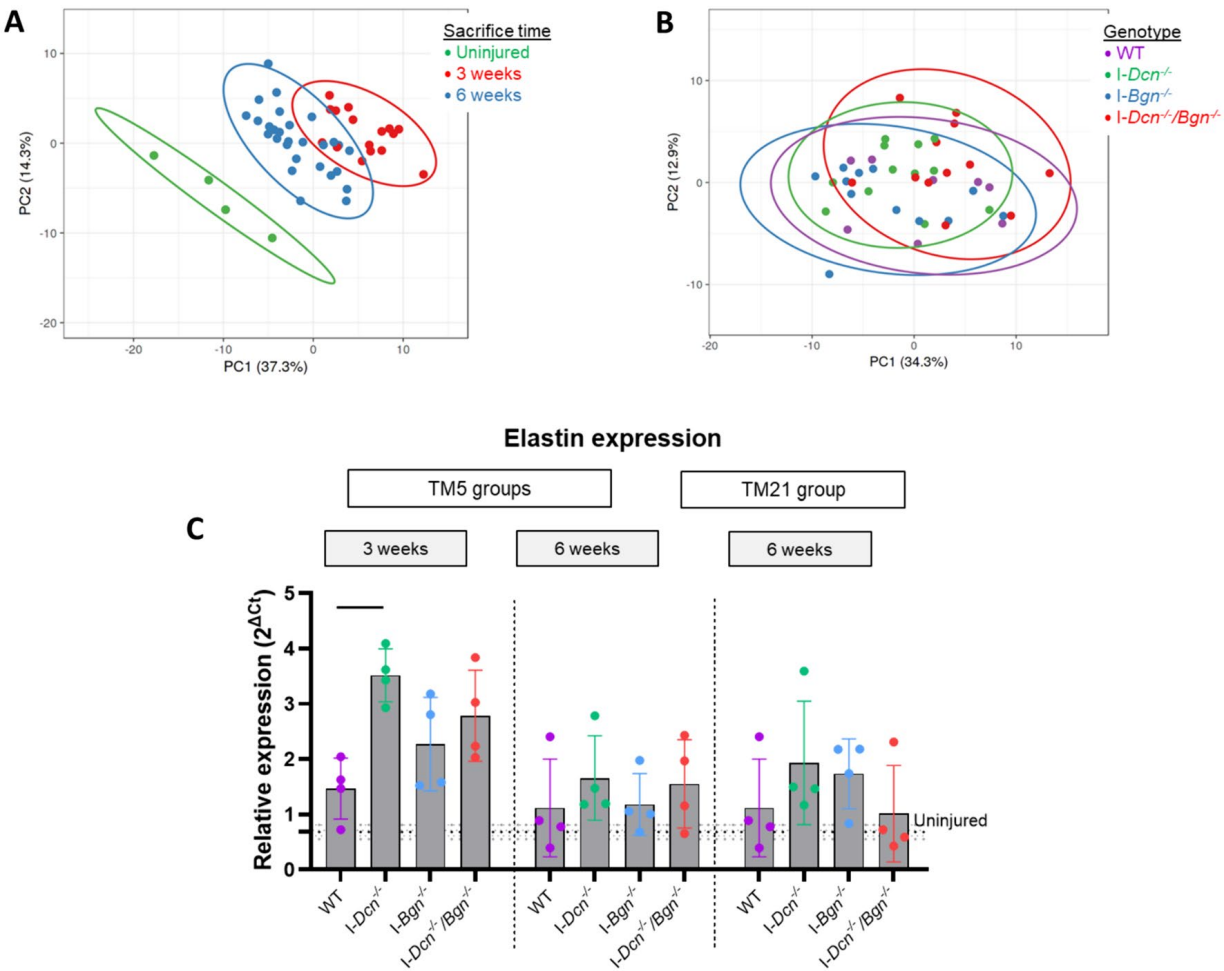
Gene expression analysis in all groups and elastin level of expression. Principal component analysis **A**–**B** showed that the major determinant of gene expression was the injury time while there was no clear separation between the genotypes (*n* = 4/group). Looking at non-collagenous matrix components, elastin expression was increased in the I-*Dcn*^*-/-*^ tendons 3 weeks post-injury (**C**). Errors bars represent standard deviation. Dotted lines represent average mRNA level of expression in uninjured tendons and grey dotted lines limits represent standard deviation of the group. Significant differences (*p* < 0.05) between genotypes are represented by solid lines.

Principal component analysis showed that injury time was the major determinant of gene expression regulation but there was no clear separation of gene expression profiles between the different genotypes (Figure 7A–B). When we looked at individual gene expression, no significant differences were detected regarding other SLRPs (fibromodulin, lumican, aspirin, keratocan), collagens (*Col1a1*, *Col1a2*, *Col2a1*, *Col3a1*, *Col5a1*, *Col5a2*, *Col6a1*, *Col6a2*, *Col11a1*, *Col12a1*, *Col14a1*), or genes involved in signaling or inflammation (T*gfb1*, *Tgfb2*, *Tgfb3*, *Tgfbr2*, *Ltbp1*, *Mtor*, *Ctgf*, *Tnf*, *Il1b*, *Il10*, *Bmp2*, *Cox-2*, *Vegfa*, *Ccl2*, *Ccl4*, *Ccl5*, *Ccl7*, *Ccl8*, *Pdgfa*, *Pgdfb*, *Pdfgra*, *Pdgfrb*, *Adgre1*, *Ptges2*, *Egr1*, *Igf1*, *Flt1*). Looking at non-collagenous matrix components (*Comp*, *Acan*, *Eln*, *Prg4*, *Vcan*, *Postn*, *Fbn1*, *Fbn2*, *Hspg2*, *Fbln4*, *Thbs2*, *Thbs4*, *Tnc*, *Tnxb*), the only observed difference was a significant increase in elastin expression at 3 weeks in the I-*Dcn*^*-/-*^ tendons (Figure 7C).

## Discussion

This study is the first to examine the temporal role of decorin and biglycan at different phases of tendon healing in the context of aging. Better understanding of the molecular mechanisms underlying the functional decline of aged tendon could provide new insights for designing new treatments to improve healing. To date, the overall effect of aging on proteoglycans is still inconclusive [20]. In tendon development, decorin and biglycan are known to regulate collagen fibrillogenesis by binding and stabilizing collagen I protofibrils to facilitate end‐to‐end and lateral growth of fibrils [9]. Contrary to our hypothesis, we did not observe more pronounced alterations of healing with decorin knockdown compared to biglycan knockdown. After knockdown, we did observe differences in fibrillogenesis as fibril diameter distributions were different between groups: at 3 weeks post-injury, the I-*Bgn*^*-/-*^ tendons exhibited more widespread fibril diameter distributions compared to both I-*Dcn*^*-/-*^ and I-*Dcn*^*-/-*^*/Bgn*^*-/-*^ tendons. In the same group, early healing appeared delayed because of a larger scar tissue area at 3 weeks, but no detectable difference remained at 6 weeks, suggesting some compensatory mechanisms.

Despite these changes in fibrillogenesis and larger scar tissue area at 3 weeks, the I-*Bgn*^*-/-*^ tendons did not exhibit a significant reduction in quasi-static or failure properties compared to WT tendons. Involvement of biglycan in early healing was suggested in previous works using biglycan-null mice [12, 13] but this could not be confirmed in the present study since recovery of mechanical properties of I-*Bgn*^*-/-*^ tendons appeared similar to WT tendons when biglycan was inactivated early. However, differences were found in viscoelastic properties between groups. Non-collagenous ECM has been suggested to contribute to tendon and ligament’s characteristic viscoelastic mechanical behavior in tension, shear, and compression [14]. The I-*Dcn*^*-/-*^ tendons exhibited a lower stress relaxation level and a higher dynamic modulus at 6 weeks when the knockdown was induced early after the injury, suggesting a better recovery of viscoelastic properties in the absence of decorin. These results contrast with those obtained with decorin transgenic null (*Dcn*^*−/−*^) aged mice, where decorin was absent throughout development, in which healing was inferior to wild-type mice [13]. In this prior study, dynamic modulus did not increase from 3 and 6 weeks after injury and tanδ was increased compared to WT suggesting an inferior ability to store and release elastic energy during cyclic loading [13]; the different results of decorin knockdown reported here are likely due to the difference in tendon development between the conventional and inducible models, highlighting the importance of using inducible models to investigate tendon healing. To determine the role of decorin in healing, other strategies to downregulate its expression have been used. The use of decorin antisense RNA in vivo led to the generation of larger diameter collagen fibrils and enhanced mechanical properties in healing ligaments [21]. In addition, engineered tendon treated with lentiviral-encoded shRNA against decorin improved healing outcomes in a rat patellar tendon model [22], this being consistent with our results. When decorin and/or biglycan knockdown was induced at a later timepoint (3 weeks post-injury), no differences were observed in tendon healing regarding the size of the scar tissue or mechanical properties suggesting a predominant impact of decorin knockdown in early healing.

Temporal roles of decorin and biglycan appeared to be different depending on age. In younger mice (120 days old), biglycan knockdown, both alone and coupled with decorin knockdown, generated a beneficial effect on tendon healing that was most pronounced at the 6 week post-injury timepoint [15]. These results confirm that the SLRPs play a different role depending on age, as our results detected a beneficial effect of decorin knockdown but not biglycan knockdown in aged mice.

However, mechanisms underlying the beneficial impact of decorin knockdown remains to be determined. Looking at gene expression level of ECM components, only an increase in elastin expression was found. Elastin is a key regulator of elastic mechanical properties in tendon and ligament [14, 23] and could contribute directly to the mechanical effects of decorin knockdown observed here. In addition, interactions have been identified between SLRPs and elastin [24] with different binding sites for decorin and biglycan in the protein core [25]. However, it is not clear how to protein interactions could affect the mechanical function in healing I-*Dcn*^*-/-*^ tendons.

The study has some limitations. First, tamoxifen‐induced Cre excision does not deplete existing decorin and/or biglycan proteins. Indeed, Dcn and Bgn expression were not fully abolished after the TMX injection (Fig. 2). In addition, Dcn and Bgn half‐life is 3 weeks or greater in the tendon ECM [26], and therefore residual SLRPS could still contribute to tendon healing after induction of knockdown. Second, we studied the impact of the knockdown on gene expression levels of other ECM components, collagens and signaling factors but did not perform protein analysis, which may follow different patterns than that of mRNA levels. The use of only female mice in this study is another limitation, and future work could investigate sex-specific roles of SLRPs in tendon healing. Additionally, because it is not possible to determine the baseline *in vivo* tension on the tendon samples, variation on how samples respond to the pre-load may have a minor impact on the strains at which viscoelastic testing was performed.

While we did not find differences in the mechanical properties, the primary healing outcome, with knockdown of biglycan and double knockdown of decorin and biglycan, it is possible that these genotypes produce a smaller effect than could be detected using our study design. However, it is questionable whether a smaller effect would be meaningful within the context of tendon healing. While the other assays had a smaller number of samples and therefore relatively larger effects may have gone undetected, genetic or structural changes that do not lead to a difference in the functional mechanics of the healing tissue are similarly not likely to be impactful.

In this study, using an inducible knockdown of decorin and/or biglycan allowed us to better understand their temporal role in tendon healing in the context of aging. Decorin and/or biglycan inactivation impacted collagen fibrillogenesis but this did not lead to detectable differences in quasi-static properties. However, early decorin knockdown appeared to be beneficial for the recovery of viscoelastic properties. Further studies will be necessary to determine how decorin regulates aged tendon healing by analyzing matrix components at the protein level, especially those binding to decorin.

## Supplementary Information

Below is the link to the electronic supplementary material.

Supplementary file1 (DOCX 495 kb)
