# Supplementary material for: Decorin Knockdown Improves Aged Tendon Healing by Enhancing Recovery of Viscoelastic Properties, While Biglycan May Not
Source: Ann Biomed Eng. 2024 Nov 29;53(3):622–33. doi: 10.1007/s10439-024-03612-y (PMC11836163; doi:10.1007/s10439-024-03612-y)

**Supplementary data**

Supplementary table 1. **Summary data of TEM analysis**. Ten images per samples were analyzed and 4 samples per group were pooled.

|  | **Minimum diameter (nm)** | **Median diameter (nm)** | **Maximum diameter (nm)** | **Interquartile range** |
| --- | --- | --- | --- | --- |
| **TM5-3 weeks** |  |  |  |  |
| WT | 17.78 | 66.48 | 212.0 | 62.47 |
| I-*Dcn^-/-^* | 14.99 | 68.49 | 177.9 | 51.67 |
| I-*Bgn^-/-^* | 19.13 | 72.87 | 168.6 | 75.19 |
| I-*Dcn^-/-^/Bgn^-/-^* | 17.51 | 75.42 | 194.2 | 56.21 |
| **TM5-6 weeks** |  |  |  |  |
| WT | 18.86 | 46.92 | 155.5 | 59.77 |
| I-*Dcn^-/-^* | 17.78 | 48.87 | 178.4 | 57.44 |
| I-*Bgn^-/-^* | 16.59 | 76.15 | 175,7 | 64.28 |
| I-*Dcn^-/-^/Bgn^-/-^* | 17.23 | 68.51 | 192.3 | 56.06 |
| **TM21** |  |  |  |  |
| WT | 18.86 | 46.92 | 155.5 | 59.77 |
| I-*Dcn^-/-^* | 19.93 | 49.39 | 197.5 | 69.12 |
| I-*Bgn^-/-^* | 18.77 | 48.84 | 187.5 | 57.17 |
| I-*Dcn^-/-^/Bgn^-/-^* | 17 | 76.65 | 185.8 | 70.43 |
| Uninjured | 19.52 | 85.49 | 169.4 | 45.26 |

Figure S1. **Pre-stamp and post-stamp cross-sectional area of the patellar tendons**. No difference was observed in cross-sectional area between groups (n=8-15/group). Errors bars represent standard deviation. Dotted lines represented average values for uninjured samples and grey dotted lines limits represent standard deviation for this group. Significant differences (p < 0.05) between genotypes are represented by solid lines. CSA : cross-sectional area.


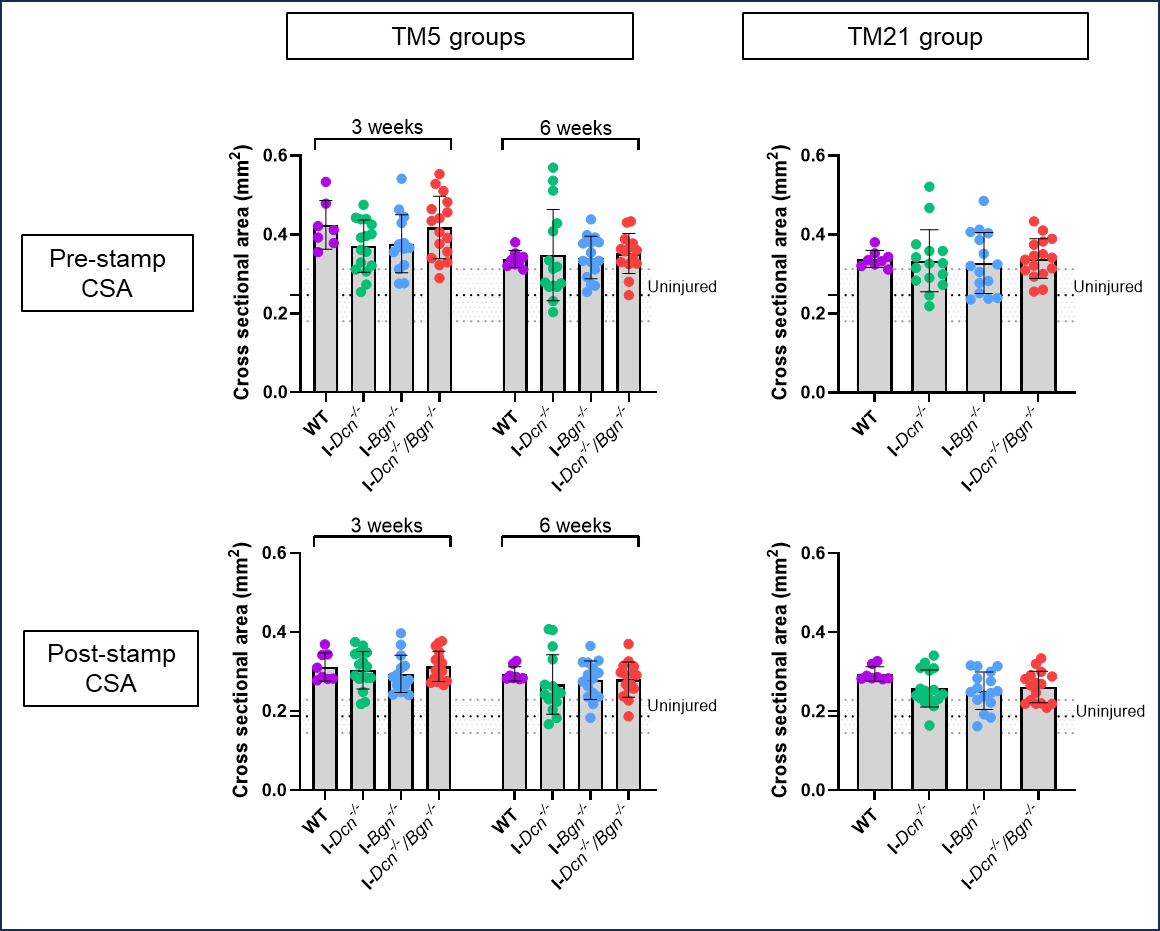


Figure S2. **Visco-elactic properties of tendons after Dcn and/or Bgn knockdown at 5 or 21 days post-injury (n=8-15/group) at 3 and 5% strain.** Decorin knockdown at 5 days post-injury was associated with a decreased stress relaxation, an increased dynamic modulus and decreased tan δ at 6 weeks compared to WT suggesting a better recovery of viscoelastic properties. Errors bars represent standard deviation. Dotted lines represented average values for uninjured samples and grey dotted lines limits represent standard deviation for this group. Significant differences (p < 0.05) between genotypes are represented by solid lines.


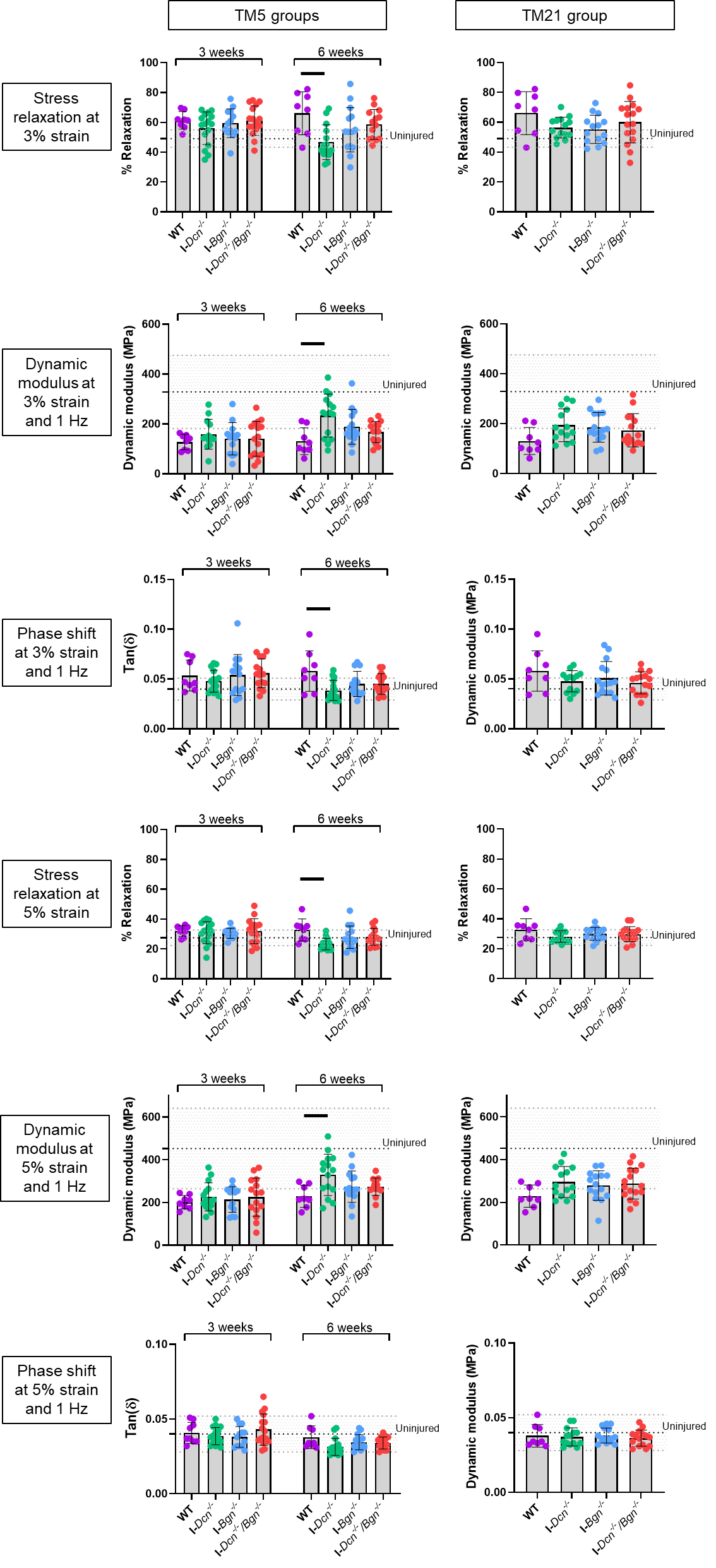

Supplement: Supplementary file 1 — Supplementary file1 (DOCX 495 kb) [file 10439_2024_3612_MOESM1_ESM.docx]
